# Genetic Characteristics, Coreceptor Usage Potential and Evolution of Nigerian HIV-1 Subtype G and CRF02_AG Isolates

**DOI:** 10.1371/journal.pone.0017865

**Published:** 2011-03-14

**Authors:** Hannah O. Ajoge, Michelle L. Gordon, Tulio de Oliveira, Taryn N. Green, Sani Ibrahim, Oladapo S. Shittu, Stephen O. Olonitola, Aliyu A. Ahmad, Thumbi Ndung'u

**Affiliations:** 1 Department of Microbiology, Ahmadu Bello University, Zaria, Nigeria; 2 HIV Pathogenesis Programme, Doris Duke Medical Research Institute, Nelson R. Mandela School of Medicine, University of KwaZulu-Natal, Durban, South Africa; 3 Nelson R. Mandela School of Medicine, Africa Centre for Health and Population Studies, University of KwaZulu-Natal, Durban, South Africa; 4 Department of Biochemistry, Ahmadu Bello University, Zaria, Nigeria; 5 Department of Gyneacology and Obstetrics, Ahmadu Bello University Teaching Hospital, Ahmadu Bello University, Zaria, Nigeria; Institute of Human Virology, United States of America

## Abstract

HIV-1 CRF02_AG and subtype G (HIV-1G) account for most HIV infections in Nigeria, but their evolutionary trends have not been well documented. To better elucidate the dynamics of the epidemic in Nigeria we characterised the *gag* and *env* genes of North-Central Nigerian HIV-1 isolates from pregnant women. Of 28 samples sequenced in both genes, the predominant clades were CRF02_AG (39%) and HIV-1G (32%). Higher predicted proportion of CXCR4-tropic (X4) HIV-1G isolates was noted compared to CRF02_AG (p = 0.007, Fisher's exact test). Phylogenetic and Bayesian analysis conducted on our sequences and all the dated available Nigerian sequences on the Los Alamos data base showed that CRF02_AG and HIV-1G entered into Nigeria through multiple entries, with presence of HIV-1G dating back to early 1980s. This study underlines the genetic complexity of the HIV-1 epidemic in Nigeria, possible subtype-specific differences in co-receptor usage, and the evolutionary trends of the predominant HIV-1 strains in Nigeria, which may have implications for the design of biomedical interventions and better understanding of the epidemic.

## Introduction

Two genetically distinct viral types of human immunodeficiency virus (HIV) are known, HIV type 1 (HIV-1) and HIV type 2 (HIV-2). HIV-1 is commonly encountered in sub-Saharan Africa, parts of Asia and Eastern Europe where HIV-1 prevalence rates are high (or growing rapidly) as well as in most other parts of the world [1], [2]. HIV-2 has been found mainly in infected individuals in West Africa and is similar to HIV-1 in its tropism for cells of the immune system and causation of illness that results from immune deficiency [1], [2]. HIV-1 variants are classified into three groups (M, O and N) and viral envelope sequences differ by up to 50% between these groups [1]. Group M is responsible for the majority of infections worldwide, and is currently classified into 13 recognised subtypes or subsubtypes (A1–A4, B, C, D, F1–F2, G, H, J, K) and 43 circulating recombinant forms (CRFs). Unlike group M, O and N viruses are restricted to west central Africa [1], [2], [3], [4].

The global prevalence of HIV-1 appears to have stabilized at 0.8%, with 33 million people living with HIV/AIDS, 2.7 million new infections, and 2.0 million AIDS deaths in 2007 [5]. The most affected region is sub-Saharan Africa, bearing 67% of the global burden [6]. The prevalence of various subtypes in West Africa is not clear, but according to recent data, 16% of the world's HIV-1 cases is in West Africa, with the dominant HIV-1 subtypes being A (21%), G (35%), CRF02_AG (28%) and other recombinants (14%; most of which is CRF06_cpx), leaving the other subtypes at less than 1% each. The same data showed that the country with by far the largest number of HIV-1 infections in the region is Nigeria, where the epidemic is dominated by subtypes A (29%) and G (54%) [6].

Nigeria is the most populous country in Africa with a population of about 140 million and a growth rate of 3.2%. Based on the national prevalence of 4.6%, it was estimated that 2.95 million people in Nigeria were living with HIV/AIDS in 2008 [7]. In 1994, partial sequencing of four HIV-1 isolates demonstrated the presence of subtype G viruses in Nigeria [8]. That same year, a new strain of HIV-1 (HIV-1 IbNg), was isolated in Ibadan, Nigeria [9]. By 1996, a full genome sequence of HIV-1 IbNg had been obtained [10], and analysis proved IbNg to be a complex mosaic genome with segments from subtype A and G, leading to the designation CRF02_AG, of which IbNg is the prototype [11]. Recent studies have shown the predominance of subtypes G and CRF02_AG in Nigeria [12], [13], [14], [15], [16]. In all, HIV-1 subtypes A, B, C, D, F2, G, J and O have been identified in Nigeria, with several recombinant forms, though in varying proportions [15], [17], .

There is some evidence that viral subtypes may have different phenotypic or clinical properties, such as coreceptor utilization, *in vitro* replication fitness, rate of disease progression, biology of transmission, antigenicity, genital shedding, drug resistance and mutational patterns [3], [4], [22], [23], [24], [25], [26]. Some of the reported differences reflect variability in the *env* gene [3], although differences have also been documented elsewhere [27], [28], [29], [30], [31]. On the other hand, the Gag protein is an important target of the immune system and cytotoxic T lymphocyte (CTL) responses targeting this protein have been shown to be associated with low viremia in some studies [32], [33], [34], [35], [36], [37].

North-Central Nigeria is one of the six geopolitical zones in Nigeria, and it consists of six out of the 36 states as well as the federal Capital Territory (FCT). As at 2005, North-Central geopolitical zone had the highest HIV prevalence [38], yet the molecular complexity has not been well documented. We thus here sequenced and analyzed the genetic characteristics of *gag* and *env* genes of HIV-1 isolates from North-Central Nigeria and searched for trends between genetic characteristics and phenotypic properties. We also used statistical and phylogenetic tools to model and estimate the origin and growth of CRF02_AG and HIV-1G in Nigeria, which to the best of our knowledge is not yet documented.

## Results

### Study population

Samples were obtained from 31 women who were part of a survey conducted in 2007 to determine the demographic attributes of and seroprevalence of HIV among pregnant women attending antenatal clinics in North-Central Nigeria. The characteristics of the population will be published elsewhere. For those analyzed for this study, briefly, the mean age was 26.8 (range, 15 to 37) years, mean age at first marriage was 21.6 (range, 12 to 31) years and all were married. Most (24) of the women were living in urban settlements. The women had at least secondary school education, except three who only had primary school education and four whose educational status is unknown. Most (17) of the women were house wives or unskilled, while the rest had skilled (defined as those with an occupation that requires at least a secondary school education or two years of professional training) or of unknown occupation. The mean number of pregnancies, abortion/miscarriages and children the women had are 2.61, 0.86 and 1.26, respectively. All women discovered their HIV status within one year of sample collection and were not on antiretroviral therapy.

### Diversity

**Figure 1:**
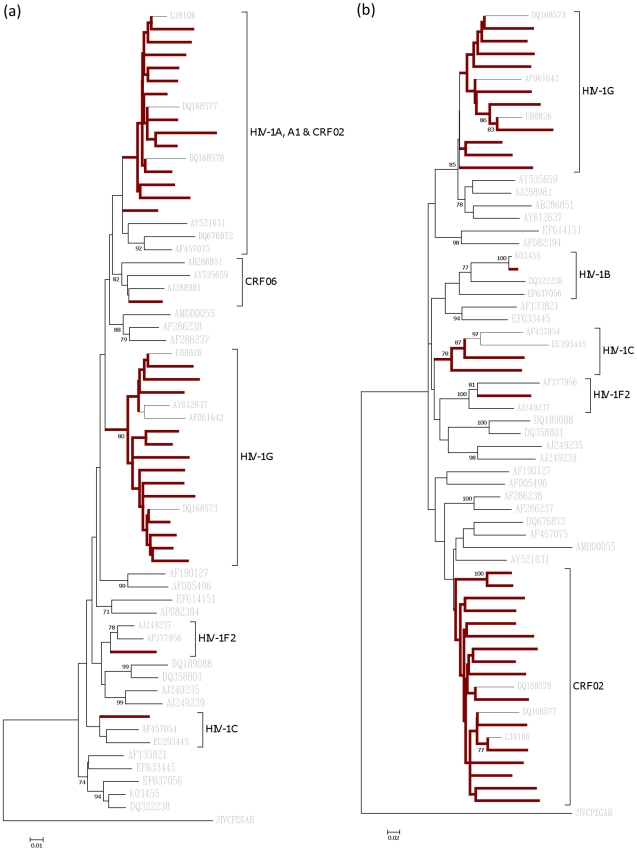
Neighbor-joining trees for (a) gag and (b) env sequences. The 29 gag and 30 env sequences were each aligned with reference sequences from the Los Alamos HIV database. The percentage (only values >70%) of replicate trees in which the associated taxa clustered together in the bootstrap test (1000 replicates) are shown. The evolutionary distances were computed using the Maximum Composite Likelihood method in Molecular Evolutionary Genetics Analysis (MEGA) software version 4.0.2. Study sequences' branches are in bold.

From the 31 samples, 29 *gag* and 30 *env* sequences were obtained. Of the 29 *gag* sequences, 13 (44.8%) were CRF02_AG, while 12 (41.4%) were HIV-1G. Also obtained was one *gag* sequence each of HIV-1C, CRF06_cpx, HIV-1F2 and a recombinant of HIV-1G and CRF02_AG. In the case of *env* sequences, 15 (50.0%) were CRF02_AG, 10 (33.3%) were HIV-1G, two were HIV-1C, one was HIV-1B, one was HIV-1F2 and one was a recombinant of CRF02_AG and HIV-1G. Neighbour-joining (NJ) tree analysis of *gag* and *env* sequences are shown in Figure 1.

To enable better phylogenetic signal, where both *gag* and *env* sequences were available, they were concatenated and subtyped. Out of the 28 samples that was sequenced in both genes, 11 (39.3%) were CRF02_AG and nine (32.1%) were HIV-1G. Other samples were one HIV-1C, one HIV-1F2 and six different types of recombinants (Table 1). All nucleotides sequences are available under GenBank accession numbers HM854072-HM854100 for *gag* sequences and HM854042-HM854071 for *env* sequences.

**Table 1:** Genetic diversity of HIV-1 isolates from antiretroviral-naive pregnant women from North-Central Nigeria.

GAG/ENV subtype	Number of Samples	%^a^	Concatenated Sequences ‘subtype’
C/C	1	3.6	C
CRF02/CRF02	11	39.3	CRF02
F2/F2	1	3.6	F2
G/G	9	32.1	G
CRF02/B	1	3.6	CRF02 B
CRF02/CRF02 G	1	3.6	CRF02
CRF06/CRF02	1	3.6	CRF02
G/C	1	3.6	G C
G/CRF02	1	3.6	G CRF02
G CRF02/CRF02	1	3.6	G CRF02
G/#	1		
#/CRFF02	1		
#/G	1		

a Percentage of the 28 samples with both gene fragments sequenced.

# Gene fragment not amplified.

### Evolutionary relationship and signature pattern of isolates from North-Central Nigeria

**Figure 2:**
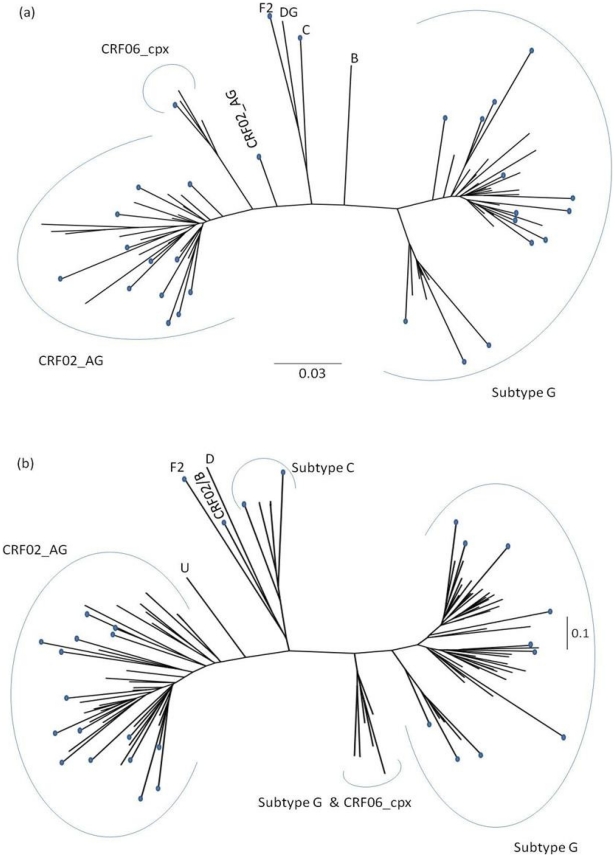
Relationship of study isolates to previous Nigerian HIV-1 isolates. Maximum likelihood trees of (a) gag and (b) env isolates (indicated with pebbles at tip) and previous Nigerian gag and env isolates (obtained from the Los Alamos HIV database), respectively, using the GTR model which was the ‘best fit’ for both data sets as determined by the FindModel tool of the Los Alamos HIV database.

*Gag* and *env* sequences were compared with all the available Nigerian sequences (corresponding to the coordinates of our sequences) downloaded from the Los Alamos HIV Database (Figure 2). The accession numbers of the downloaded sequences are listed in Table S1. Using maximum likelihood methods, both *gag* and *env* sequences intermingled with the available Nigerian *gag* and *env* sequences. The CRF02_AG and HIV-1G clusters were distinct, with the HIV-1G cluster consisting of two distinct sub-clusters in the *gag* tree and three distinct sub-clusters in *env* tree. In the *env* tree one of the three sub-clusters consisted of intermingled HIV-1G and CRF06_cpx sequences, unlike in the *gag* tree where CRF06_cpx sequences existed as a distinct clade. Prior to now only one HIV-1F2 sequence has been documented in Nigeria [13], [21]. The identified HIV-1F sequence from the current study was compared to the few available sequences from the Los Alamos HIV database, using maximum likelihood method. The isolate clustered in both genes with HIV-1F2 isolates from Cameroon (data not shown).

**Figure 3:**
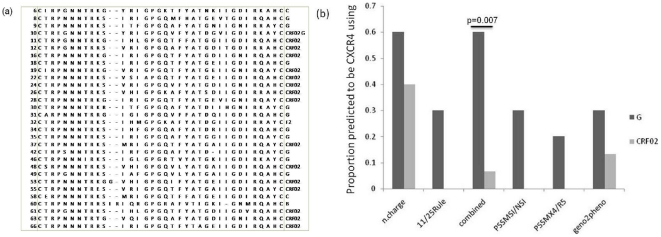
Env V3 amino acid sequences and CXCR4 usage potential of isolates. (a) Env V3 amino acid sequences; each sequence is flanked by its sample number on the left and the subtype on the right. (b) A chart of relative CXCR4 usage prediction based on net charge rule [n.charge], the 11/25 rule, the combined criteria from the 11/25 and net charge rules [combined] as described by Raymond et al (2009), the web-based specific position-specific scoring matrixes (PSSM) programme and the web-based Geno2pheno tool.

Signature patterns were searched for between present isolates and the previous Nigerian isolates, in order to identify possible genetic features distinguishing isolates from this study compared to those from earlier studies. There was no difference between the signature patterns of study CRF02_AG *gag* sequences and previous isolates. However, with respect to HIV-1G, there were differences at HXB2 amino acids' coordinates 158, 172, 214, 247, 285 and 314. In the case of *env*, we focused on the V3 loop due to its crucial role in coreceptor usage. There was only one difference in CRF02_AG sequences at position five of the V3 loop, an asparagine in 80% of current study sequences compared to 56% glycine for previous sequences. Two amino acid differences were noted for HIV-1G sequences in the 14^th^ and 34^th^ V3 loop positions, with phenylalanine and tyrosine in the study V3 loop sequences at a frequency of 0.50 each; while isoleucine and histidine were present in the previous sequences at frequencies of 0.85 and 0.75, respectively. The *env* V3 loop amino acid sequences are shown in Figure 3a.

### Genetic characteristics of isolates from North-Central Nigeria

The main purpose of sequencing *gag* gene was to corroborate *env* gene sequence data for the subtyping of isolates given previous reports of complex diversity of HIV-1 isolates in Nigeria. We also investigated characteristic sequence differences that may distinguish between the CRF02_AG and HIV-1G *gag* sequences in this study. However, the only amino acid characteristic of note was the presence of the H219Q substitution, which confers the greatest replication potential to HIV-1 variants resistant to protease inhibitors [39], [40], in two (15.39%) of CRF02_AG and all the HIV-1G isolates. The H219Q substitution seems to be a polymorphism in HIV-1G as all previous isolates from Nigeria and 93.8% worldwide harbour the substitution [21].

Coreceptor usage potential of isolates was predicted using the net charge rule, the 11/25 rule, the combined criteria from the 11/25 and net charge rules as described by Raymond *et al* [41], Geno2pheno web-based tool and specific position-specific scoring matrix (PSSM) programme (also a web-based tool). Consistent with previous observations [3], all the five methods predicted CRF02_AG to be predominantly CCR5-using. There is no previous data on HIV-1G tropism, thus it was interesting to note that all the five methods showed a trend toward higher usage of CXCR4 in HIV-1G in comparison to CRF02_AG and individually, was found to be statistically significant (p = 0.007, Fisher's exact test) using the combined criterion (Figure 3b), which has previously been shown to perform best for the prediction of CRF02_AG coreceptor usage [41].

The tetrapeptide motif on the tip of the V3 loop of *env* varies among subtypes and this variability may have biological importance [3], [42]. Most (80%) of the isolates had the GPGQ amino acid motif at the V3 loop, other motif sequences were APGQ, GPGK and GPGR. As expected, the subtype B isolate had the GPGR motif and non-B isolates predicted to be CCR5-using (using the combined rule criteria) were significantly more likely to have the GPGQ motif than those predicted to be CXCR4-using (p = 0.02, Fisher's exact test).

Mutations in the V3 loop appear to play a key role in conferring CCR5-tropic maraviroc-resistant phenotype [43]. None of our 20 predicted (combined criteria rule) CCR5-tropic sequences had the combinations of mutations which has been previously described and found in 7% of maraviroc-naive viruses [44], [45], [46].

### Origin and demographic history of Nigerian CRF02_AG and HIV-1G

**Figure 4:**
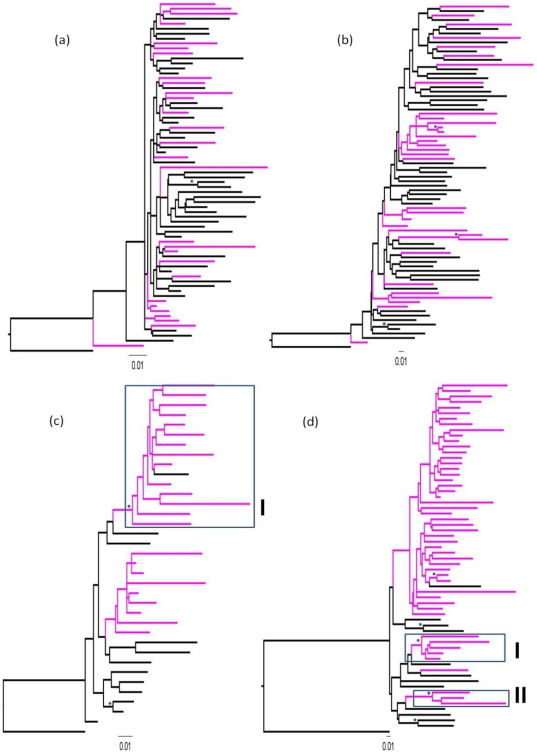
Relationship between Nigerian isolates and reference sequences from other parts of the World. Maximum likelihood analysis of (a) CFR02_AG gag, (b) CFR02_AG env, (c) HIV-1G gag and (d) HIV-1G env sequences. Each tree shows the relationship between Nigerian sequences (purple) and reference sequences from other countries (black), using the GTR best model as determined by FindModel tool of the Los Alamos HIV database. Trees were rooted using HIV-1B (HXB2) as outgroup. Bootstrap resamplings (1000 replicas) was used to assess robustness and values ≥70 are indicated with asterisk.

To investigate the origin of CRF02_AG and HIV-1G in Nigeria, we compiled data sets of Nigerian and reference sequences for these subtypes from different geographic origins for both *env* and *gag* regions under study. The Nigerian sequences were derived from all of the available sequences (Table S1) in the Los Alamos HIV database corresponding to our region of interest (corresponding HXB2 coordinates 1234–1833 for *gag* data sets and 7068–7589 for *env* data sets), as well as those obtained from this study that were subtype concordant in both regions. The CRF02_AG *gag* and *env* data sets consisted of 28 and 33 sequences respectively, with both data sequences dating from 1994 to 2007, while the HIV-1G *gag* and *env* data sets consisted of 23 and 50 sequences respectively, with both data sequences dating from 1992 to 2007. The references sequences (Table S1) from other countries were downloaded from the HIV Los Alamos data base by accessing the HIV Sequence Alignments page of the database, and downloading 2008 HIV-1 subtype reference of corresponding to the HXB2 coordinates of the appropriate gene fragment. Maximum likelihood phylogenetic analysis was carried out to elucidate the relationship. Trees were rooted using HIV-1B (HXB2) as outgroup. For clarity, tip labels are removed and the branches for the Nigerian isolates are coloured in purple and the reference sequences which are from other different countries are in black. In the *gag* and *env* trees (Figure 4a and b), the Nigerian CRF02_AG sequences were dispersed among other sequences from other countries without any well-supported clade, suggesting multiple independent entries of this subtype into Nigeria. The Nigerian HIV-1G were also dispersed among other sequences from other countries, suggesting that HIV-1G also made multiple independent entries into Nigeria, however, they had one and two well-supported clades (bootstrap ≥70%) in the *gag* and *env* trees respectively (Figure 4c and d).

Bayesian methods for estimation of phylogeny under both strict and uncorrelated lognormal molecular clock models was performed on *gag and env* gene fragments of Nigerian HIV-1G using Beast v1.5.3 [47]. Nigerian CRF02_AG had no well-supported clade in the phylogenetic analysis and thus did not warrant further analysis. The constant population size, exponential growth as well as nonparametric Bayesian skyline plot tree priors were used for inference. Regardless of the model involved, the two independent runs of the Markov chain yielded similar results. Based on twice the natural logarithm of Bayes factor [48], [49], models enforcing a relaxed clock over a strict clock were supported, regardless of gene or subtype. Under the relaxed clock, the Bayesian skyline plot and the constant population size were both supported (the alternative model was not strongly supported over the null model), thus the two models were adopted for making inferences. Models' natural logarithm of Bayes factor is shown in Table 2. It is noteworthy that the exponential growth models did not fit the data sets, as indicated by effective sample size (ESS) scores <200 (data not shown). All models used had ESS greater than 600.

**Table 2:** Natural logarithm Bayes factors between different models.

Data set	H1/H0	BSP relaxed	CS relaxed	BS strict	CS strict
HIV-1G GAG	BSP relaxed	-	−1.337	4.903	4.776
	CS relaxed	1.337	-	6.24	6.113
	BS strict	−4.903	−6.24	-	−0.127
	CS strict	−4.776	−6.113	0.127	-
HIV-1G ENV	BSP relaxed	-	−2.089	7.582	6.608
	CS relaxed	2.089	-	9.671	8.698
	BS strict	−7.582	−9.671	-	−0.973
	CS strict	−6.608	−8.698	0.973	-

BS: Bayesian Skyline plot; CS: constant population size; relaxed: relaxed molecular clock; strict: strict molecular clock Evidence against H0 (null) model is assessed in the following way; 0–6 indicates positive evidence for H1 (alternative) model, >6 indicates strong evidence for H1. The H0 models are in the first row while the H1 models are in the first column.

**Figure 5:**
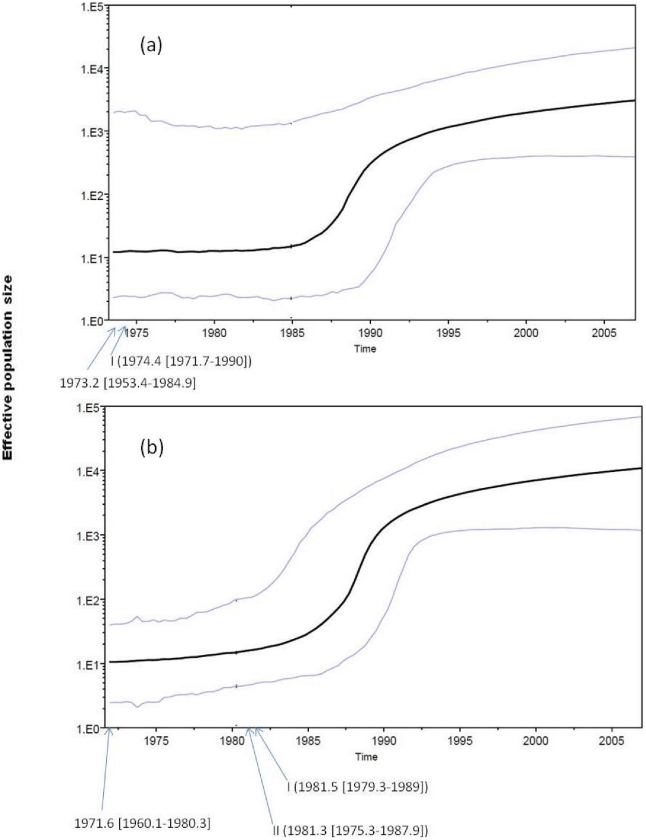
Bayesian skyline plots of Nigerian HIV-1G. Past population dynamics of HIV-1G [based on (a) *gag* and (b) *env* data sets] infections reconstructed by Bayesian skyline plot. The first arrow from the left indicates the time of the MRCA. The other arrows indicate the estimated origin of the corresponding Nigerian clade (indicated by the Roman numeral) in the trees in Figure 4 c and d. HPDs (95%) are given in parenthesis beside each estimate.

Based on the *gag* data set, the relaxed Bayesian skyline plot and the relaxed constant population size models estimated the Nigerian HIV-1G's most recent common ancestor (MRCA) to 1973.2 [HPDs 1953.4–1984.9] and 1957.3 [HPDs 1867.4–1982.9], respectively. Still with both models, based on the *env* data set, the MRCA was traced to 1971.6 [1960.1–1980.3] and 1969.5 [HPDs 1955.6–1979.3], respectively. Date of the most common recent ancestor and parameters estimated for each model are shown in Table S2, while the evolutionary rate and parameters estimated for each model are in Table S3. Due to the likely scenario of multiple independent entries (from neighbouring countries) caution should be taken in the interpretation, as these most recent ancestors is not necessarily the ancestor of Nigerian isolates alone nor a reflection of how early the subtypes has been in the country. Still with the Bayesian analysis the origin of the Nigerian transmission clades observed in figure 4 c and d was traced to 1974 with the gag data set and 1981 with the *env* data set (Figure 5). Thus we could speculate the possible presence of the subtype in the country by or earlier than the early 1980s, involving a three phase growth. The three phase growth consists of a pre-1985 constant population, an exponential growth from 1985 to early 1990s, and a slower post early 1990 growth. But these speculations by the Bayesian skyline plot needs confirmation through the demonstration of these subtypes in archived samples, as there could be several possible explanations since we are not dealing with a closed population, more so since the constant population demographic model cannot be rejected.

## Discussion

Nigeria is the home to the second largest population of people living with HIV/AIDS, with this large number of infected people, routine and systematic surveillance of the genetic diversity of HIV is necessary. Thus our carrying out an exploratory study to characterize *gag* and *env* signature sequences associated with the epidemic in Nigeria was in order, especially with the need for new/novel therapeutic and prophylactic strategies that target these important genes. Furthermore, a detailed characterization of the introduction and growth of HIV-1 in Nigeria is lacking and relevant data could be useful for designing strategies to prevent the spread of HIV-1 and future epidemics. Hence we explored the genetic variability of HIV-1 in North-Central Nigeria which is the geopolitical zone with the highest HIV prevalence rate [38], and also explored the temporal genetic variation of Nigerian *gag* and *env* genes of CRF02_AG and HIV-1G.

Consistent with data from previous studies in Nigeria from other regions [12], [13], [14], [15], [16], we observed that the HIV epidemic in North-Central Nigeria is driven predominantly by CRF02_AG and HIV-1G. Of 28 samples sequenced in *gag* and *env* genes, 6 (21%) had discordant subtypes between the two genomic regions, as observed with recent studies in Nigeria [16], [20]. These discordant samples may represent new recombinant forms or coinfection/superinfection (which could ultimately also result in the formation of unique recombinant forms). Based on the relatively small gene fragments we sequenced, we cannot make an inference as to the true nature of these discordant samples. Over 20% of the current HIV-1 infections in Africa are estimated to represent recombinant strains, and yet the possible consequences of the emergence of recombinant HIV-1 strains is not yet understood [50]. Beside CRF02 and HIV-1G, we isolated other subtypes, all of which have long been known to exist in Nigeria, though to a relatively low prevalence. Thus the complexity of the HIV-1 pandemic in Nigeria may still be well underrepresented.

Using Maximum likelihood method we observed that our isolates at both *gag* and *env* genes intermingled with previous Nigerian ones with no founder effect noted. The signature pattern analysis also showed similarities between our isolates and previous Nigerian isolates. Subtype G radiation (sub-clustering) was seen in both the *gag* and *env* trees as observed previously [15], [16], [20], [51]. Because the sub-cluster corresponding to the prototypic subtype G constitute minority of the sequences, the sub-cluster containing majority of the subtype G sequences was classified as G′ [15], [20], [51]. The relationship of this “Nigerian subtype G” (denoted as G′) to the prototypical subtype G for future immunological and vaccine research in Nigeria, may only be assessable through full-length genome amplification [16]. The HIV-1F2 isolate noted in this study is only the second isolate of this clade from Nigeria. It is interesting to note that the first isolate was from Jos which is a city in North-Central Nigeria [13], [21]. This sequence closely clusters with Cameroonian sequences and might have been introduced into Nigeria from Cameroon. This is not surprising as Cameroon shares a border with Nigeria and HIV-1F2 is presumed to have originated in Cameroon [52].

The H219Q substitution in the cyclophilin A (CypA) binding loop in *gag* seen in all Nigerian HIV-1G and 93% of all isolates suggests that it is a naturally occurring polymorphism in this subtype. In the development of high level HIV-1 resistance against protease inhibitors, H219Q substitution in Gag emerges earlier than substitutions in protease [39], and has been shown to confer the greatest replication potential to HIV-1 variants resistant to protease inhibitors [40]. It has been observed that there is naturally occurring decreased susceptibility of HIV-1G to protease inhibitors [53]. It is possible that the H219Q polymorphism in HIV-1G may contribute to this observation. We also noticed other polymorphisms in the CypA binding loop at positions 218 and 222 that may have unknown effect on CypA binding and viral replication. Though the CypA binding loop of HIV-1B has been modelled [40], there is a need for an in-depth molecular modelling of the CypA binding loop of HIV-1G, which seems to be different from HIV-1B. Furthermore, the effectiveness of protease inhibitors in geographic areas where CRF02_AG and HIV-1G predominate may require careful monitoring as our data suggests that these subtypes may have lower genetic barrier for an important compensatory mutation in *gag* once protease resistance emerges.

Along with CD4, HIV-1 requires a chemokine receptor, CCR5 or CXCR4, as an entry co-receptor, and differential coreceptor selectivity is an important determinant of viral diversity and pathogenesis. Besides, the interest in HIV tropism has recently risen with the development of CCR5 antagonists as clinically applicable anti-HIV agents [54], [55], [56]. Though the “gold standard” for characterization of HIV-1 tropism is a recombinant virus phenotypic entry assay, genotypic methods based on the V3 sequence have been successful with HIV-1B, and could be an easier and more cost effective option and might prove useful as screening strategy in clinical practice [54], [57]. There has been more success in the determination of tropism of non-B viruses as a result of modifications/improvements in prediction rules and bioinformatic tools [41], [58]. Our prediction on CRF02_AG isolates is consistent with previous observation [3], and thus shows that individuals infected with CRF02_AG are likely to benefit from coreceptor antagonists like maraviroc, though high CXCR4 usage has been observed in late infections involving CRF02_AG [59]. However, individuals infected with HIV1-G may not equally benefit since all the five methods predicted higher usage of CXCR4 in HIV-1G in comparison to CRF02_AG, especially the combined criterion which has previously been shown to best predict CRF02_AG coreceptor usage [41], [59]. Our study thus highlights a need for better characterization of HIV-1G tropism, because more data is clearly needed, especially since HIV-1G makes up 6.3% of the global HIV infections [60]. Nevertheless, our study provides evidence that this subtype may have a higher proportion of X4 variants compared to CRF02_AG and other non-B subtypes although phenotypic characterization is clearly warranted.

Two known mutational pathways of viral escape from CCR5 antagonists are selection of R5X4/X4-tropic viruses or development of resistance to the antagonists [61]. None of the predicted CCR5-using isolates from this study had the combination of mutations that confer resistance to coreceptor antagonists, which suggests that maraviroc will be relevant in the management of CCR5-using HIV-1 in Nigeria. However, coreceptor usage ability of patients' viral population will clearly be required before clinical administration, especially with the significant presence of HIV-1G which by our study shows to have higher potential for CXCR4 usage.

Phylogenetic inference from molecular sequences is becoming an increasingly popular tool to trace the patterns of pathogen dispersal, especially for rapidly evolving RNA viruses [62], [63], [64], [65]. Here we utilized a combination of maximum likelihood and Bayesian methods to decipher the epidemiology and evolution of the predominant HIV-1 clades in Nigeria from sampled gene sequence data. The exponential models did not the fit our data sets which could be due to insufficient information in the data to accommodate a complex tree model, as has been observed previously [66], or because the Nigeria epidemic cannot be explained using an exponential growth model. We found the Bayesian skyline plot a useful method for estimating ancestral population dynamics from our sample of molecular sequences as shown previously [67]. The approach showed that both clades made multiple entrances into the country to establish the present day epidemic, similar to subtype C in Zimbabwe [64], and HIV-1G probably had been in the country since the early 1980's or earlier. These findings are consistent with what is known about HIV-1 origin in Nigeria, as the first HIV positive person was identified in 1983 [38]. Based on the shape of the skyline plots, we could extrapolate that HIV-1G in Nigeria, had a three phase growth. The first phase involved a pre-mid 80s constant population, fast growth for about 9 years (from around 1985 to 1993) and a much slower phase from around 1993 to 2007. However, the error intervals (HPDs) especially with *gag* data set and Bayes factor do not allow us to exclude that, in average, the epidemic in Nigeria has been constant over time, which can even suggest a decrease in the last decade. Since HIV-1G constitute substantial part of the overall HIV-1 epidemic in Nigeria, the overall growth of HIV-1 may be similar. Thus it is noteworthy that we observed that the growth rate became slower from around 1993, which is the year in which the present Nigerian democracy began. It may be possible that since 1993, as a result of democracy and increased socio-political stability and increase in media awareness, the HIV-1 growth rate declined. Our study, not unlike studies in other countries, suggests that complex socio-political and economic changes can dramatically influence the epidemiology of a newly introduced pathogen in a population [64], [68]. Though both the *gag* and *env* data sets produced results that are corroborative, as shown by the Bayesian skyline plots (Figure 5), we still need to produce a more detailed sampling in order to capture the earlier and more recent population demography. The lack of resolution (and high HPDs) especially with *gag* data set is most likely due to the fact that the sample used is small, both in sequence size as well in temporal distribution.

In conclusion our study showed that there is a high genetic complexity of HIV-1 in North-Central Nigeria which is predominated by CRF02_AG and HIV-1G. We observed apparent differences in *gag* with respect to a mutation that could confer increased replication capacity in the presence of protease inhibitor resistance mutations, potential differences in coreceptor usage and lack of naturally occurring mutations that confer resistance to CCR5 antagonists. Lastly we showed that Nigerian HIV-1 demography involved multiple introductions, a phase of early growth (pre-democracy) and phase of stabilisation (post-democracy) or even decrease of the HIV epidemic. Our observation on genetic complexity may be limited by the relatively small numbers of samples sequenced and small fragments analyzed rather than full length genomes. Also the observation on coreceptor usage is limited by the absence of phenotypic/functional assays, patients' CD4 counts and clinical staging of the infection in the current study. Furthermore, the scarcity of Nigerian HIV-1 sequences limits the analytic and predictive power of the Bayesian and phylogenetic methods used. We therefore recommend phenotypic assay based study on HIV-1G tropism as well as more surveillance studies involving archive and recent strains and full-length genome sequencing in order to better understand HIV diversity in Nigeria.

## Materials and Methods

### Ethics statement

The institutional ethics review board of the Ahmadu Bello University Teaching Hospital, Zaria, Nigeria and the ethics boards/committees of the various facilities involved approved the study and informed consent was received from each participant.

### Study population

The samples sequenced in this study was derived from a survey conducted from August to November 2007 to determine the demographic attributes and seroprevalence of HIV among therapy-naïve pregnant women attending antenatal clinics of four health facilities in North-Central Nigeria. The facilities involved were: Federal Medical Centre, Markudi in Benue; Bwarri General Hospital, in FCT; General Hospital, Minna in Niger; and Church of Christ in Nigeria (COCIN) Community Development Program [CCDP] Hospital, Panyam in Plateau. Relevant primary study information on the survey will be published elsewhere.

### RNA extraction, reverse transcription, and PCR amplification of gag

RNA was extracted from plasma using the QIAamp Viral RNA mini kit (Qiagen, Heiden, Germany) according to the manufacturer's instructions. The RNA template and 100 ng of primers (random hexamers) were heated at 65°C for 5 min, chilled at 4°C for 1 min and reverse transcribed at 50°C for 60 min, followed by 55°C for 60 min and finally held at 70°C for 15 min in a 20 µl reaction volume containing, 5× reaction buffer, 10 mM dithiothreitol, 0.5 mM each deoxynucleoside triphosphate, 40 U RNase OUT (Invitrogen) and 200 U Superscript III reverse transcriptase (Invitrogen). RNaseH (Invitrogen) was subsequently added to the reaction and incubated at 37°C for 20 min. The cDNA was stored at −20°C until needed.

The p24 *gag* region was amplified from cDNA with G00 (5′-GACTAGCGGAGGCTAGAAG-3′; positions 764 to 782, according to HxB2 coordinates [69] and G01 (5′-AGGGGTCGTTGCCAAAGA-3′; positions 2,264 to 2,281) as outer primers. G25 (5′-ATTGCTTCAGCCAAAACTCTTGC-3′; positions 1,867 to 1,889) and G60del3′G (5′- CAGCCAAAATTACCCTATAGTGCA-3′; positions 1,173 to 1,197) were the inner primers. The PCR conditions for both the first and second round reactions were initial denaturation at 95°C for 10 min, followed by 35 cycles at 94°C for 30 s, 45°C for 35 s, and 72°C for 60 s.

### RNA extraction, reverse transcription, PCR amplification and cloning of env

RNA extraction, and reverse transcription were carried out as for the *gag* gene, except that primer OFM-19 [70] was used. The PCR conditions were as described previously [71].

Cloning was modified from Singh *et al* [72], using the TOPO TA cloning Kit (with pCR2.1-TOPO vector) with One Shot TOP10 chemically competent *E. coli* (Invitrogen).

### Visualisation of amplified products and sequencing and sequence analysis

Amplified products were visualised on 1% agarose gels and PCR products or clones sequenced using the inner primers as described previously [72]. Sequences were assembled and edited with Sequencher 4.8 and aligned with reference sequences obtained from Los Alamos HIV database using Bioedit 5.0.9 [73], with manual editing as necessary. The aligned trimmed products of the *gag* region corresponds to positions 1,234 to 1,833 (600 bp; Gag residues 149 to 348), while aligned trimmed products of the *env* region corresponds to positions 7,068 to 7,616 (549 bp; C2–C4). Phylogenetic analyses were conducted using the Maximum Composite Likelihood method in Molecular Evolutionary Genetics Analysis (MEGA) software version 4.0.2 [74] and bootstrap consensus trees were inferred from 1,000 replicates. To identify recombinants, analysis was performed on the *gag*, *env*, and concatenated sequences (where samples were amplified on both genes), using a combination of Recombinant Identification Program, RIP 3.0 from the Los Alamos HIV database and SimPlot version 3.5.1 [75]. Recombinants were further confirmed with Neighbor-Joining (NJ) trees using the estimated breakpoint obtained from the SimPlot analysis.

Differences in signature patterns between samples in this study and those from previous ones in Nigeria were identified using Viral Epidemiology Signature Pattern Analysis (VESPA) software [76], [77]. Coreceptor utilization was predicted using three rules and two web-based bioinformatics tools. The rules were the net charge rule [78], the 11/25 rule [79] and the combined criteria from the 11/25 and net charge rules as described by Raymond *et al* [41]. The web-based tools used were the Geno2pheno [80] and position-specific scoring matrix (PSSM) programme [58], [81]. Graphical representation of conserved amino acids was done using a web-based sequence depiction tool, WebLogo 3.0 software [82].

### Evolutionary analysis

The best fitting nucleotide substitution model was evaluated using a web-based tool, FindModel [83]. The model chosen for the alignments was the GTR (general time reversible model). Maximum likelihood (ML) phylogenies were estimated for each dataset under the model, starting with a NJ starting tree and using SPR (subtree pruning and regrafting) heuristic search algorithms. Calculations were performed with Phylogenetic Analysis Using Parsimony (PAUP*) 4.0b10 written by David L. Swofford. Statistical support for ML phylogeny structures was evaluated by bootstrapping analysis of the original sequence alignments (1,000 NJ replicates). Trees were rooted by outgroup (HxB2) and presented using the program FigTree 1.3.1 written by Andrew Rambaut. Bayesian estimates of phylogeny were obtained using BEAST v1.5.3 [47], under both strict and relaxed lognormal molecular clock models. All analysis was performed under the HKY nucleotide substitution model and SRD06 model used for partitioning into codon positions. Two parametric models (constant population and exponential growth) and one nonparametric model (Bayesian skyline) tree priors were used for the inference. For each model, two independent runs of the Markov chain were performed. For the *gag* gene, each Markov chain Monte Carlo (MCMC) run was 20,000,000 steps long, while for the *env* gene, MCMC runs were 30,000,000. Both were sampled every 10,000 steps. The Effective Sampling Size (ESS) was calculated by combining the output of the two runs (that both converged and mixed properly) using BEAST v1.5.3's Log Combiner, excluding an initial burn-in of 10% for each chain and viewed in Tracer v1.5.0 (written by DRUMMOND, A. J. and RAMBAUT, A.). The ESS value of >200 was taken as a sufficient level of sampling. Model comparison was achieved by calculating Bayes factor [48].

### Statistical analysis

To analyse difference in predicted CXCR4 usage of CRF02_AG and HIV-1G, the results of the coreceptor predictions according to each rule/tool were analysed for differences using Fisher's exact test, as implemented in GraphPad Prism version 5.00 for Windows, GraphPad Software, San Diego California USA, www.graphpad.com.

## Supporting Information

Table S1 **Accession number of sequences downloaded from the HIV Los Alamos Database.**
(DOC)

Table S2 **Date of Most Common Recent Ancestor (MRCA) and parameters estimated from BEAST.**
(DOC)

Table S3 **Evolutionary rate and parameters estimated from BEAST.**
(DOC)
