# Supplementary material for: Genetic Characteristics, Coreceptor Usage Potential and Evolution of Nigerian HIV-1 Subtype G and CRF02_AG Isolates
Source: PLoS One. 2011 Mar 14;6(3):e17865. doi: 10.1371/journal.pone.0017865 (PMC3056731; doi:10.1371/journal.pone.0017865)
Supplement: Table S1 — Accession number of sequences downloaded from the HIV Los Alamos Database. (DOC) [file pone.0017865.s001.doc]

**Table S**1. Accession number of sequences downloaded from the HIV Los Alamos Database.

| **Subtype** | **Nigerian gag sequences*** | **Nigerian env sequences*** | **Dated Nigerian gag sequences**** | **Dated Nigerian env sequences**** | **Reference gag sequences***** | **Reference env sequences***** |
| --- | --- | --- | --- | --- | --- | --- |
| CRF02_AG | Top of Form  [AJ269982](http://www.hiv.lanl.gov/components/sequence/HIV/asearch/query_one.comp?se_id=149258) Bottom of Form | Top of Form  [DD409979](http://www.hiv.lanl.gov/components/sequence/HIV/asearch/query_one.comp?se_id=81918) | AJ269982 | Top of Form  [AF069933](http://www.hiv.lanl.gov/components/sequence/HIV/asearch/query_one.comp?se_id=233537)Bottom of Form | CM.AY371122 | CM.AY371122 |
|  | Top of Form  [AJ269987](http://www.hiv.lanl.gov/components/sequence/HIV/asearch/query_one.comp?se_id=149256) Bottom of Form | Top of Form  [AF069933](http://www.hiv.lanl.gov/components/sequence/HIV/asearch/query_one.comp?se_id=233537) | AJ269987 | Top of Form  [AF069941](http://www.hiv.lanl.gov/components/sequence/HIV/asearch/query_one.comp?se_id=233529) | CM.AY371123 | CM.AY371123 |
|  | Top of Form  [AJ269988](http://www.hiv.lanl.gov/components/sequence/HIV/asearch/query_one.comp?se_id=149255) Bottom of Form | Top of Form  [AF069941](http://www.hiv.lanl.gov/components/sequence/HIV/asearch/query_one.comp?se_id=233529)Bottom of Form | AJ269988 | Top of Form  [AJ389758](http://www.hiv.lanl.gov/components/sequence/HIV/asearch/query_one.comp?se_id=226286) Bottom of Form | CM.AY371124 | CM.AY371124 |
|  | Top of Form  [AJ269992](http://www.hiv.lanl.gov/components/sequence/HIV/asearch/query_one.comp?se_id=149253) Bottom of Form | Top of Form  [AJ389758](http://www.hiv.lanl.gov/components/sequence/HIV/asearch/query_one.comp?se_id=226286)Bottom of Form | AJ269992 | Top of Form  [AJ389759](http://www.hiv.lanl.gov/components/sequence/HIV/asearch/query_one.comp?se_id=226286)Bottom of Form | CM.AY371131 | CM.AY371131 |
|  | Top of Form  [AJ269993](http://www.hiv.lanl.gov/components/sequence/HIV/asearch/query_one.comp?se_id=149252) Bottom of Form | Top of Form  [AJ389759](http://www.hiv.lanl.gov/components/sequence/HIV/asearch/query_one.comp?se_id=226286) Bottom of Form | AJ269993 | Top of Form  [AJ389760](http://www.hiv.lanl.gov/components/sequence/HIV/asearch/query_one.comp?se_id=226286)Bottom of Form | CM.AY371137 | CM.AY371137 |
|  | Top of Form  [AJ269997](http://www.hiv.lanl.gov/components/sequence/HIV/asearch/query_one.comp?se_id=149249) Bottom of Form | Top of Form  [AJ389760](http://www.hiv.lanl.gov/components/sequence/HIV/asearch/query_one.comp?se_id=226286)Bottom of Form | AJ269997 | Top of Form  [AJ389761](http://www.hiv.lanl.gov/components/sequence/HIV/asearch/query_one.comp?se_id=226286)Bottom of Form | CM.AY371132 | CM.AY371132 |
|  | Top of Form  [AJ269998](http://www.hiv.lanl.gov/components/sequence/HIV/asearch/query_one.comp?se_id=149248)Bottom of Form | Top of Form  [AJ389761](http://www.hiv.lanl.gov/components/sequence/HIV/asearch/query_one.comp?se_id=226286) Bottom of Form | AJ269998 | Top of Form  [AJ389763](http://www.hiv.lanl.gov/components/sequence/HIV/asearch/query_one.comp?se_id=226286)Bottom of Form | CM.AY371134 | CM.AY371134 |
|  | Top of Form  [AJ269999](http://www.hiv.lanl.gov/components/sequence/HIV/asearch/query_one.comp?se_id=226328)Bottom of Form | Top of Form  [AJ389763](http://www.hiv.lanl.gov/components/sequence/HIV/asearch/query_one.comp?se_id=226286) Bottom of Form | AJ269999 | Top of Form  [AJ389764](http://www.hiv.lanl.gov/components/sequence/HIV/asearch/query_one.comp?se_id=226286) Bottom of Form | CM.AY371136 | CM.AY371136 |
|  | Top of Form  [AJ270000](http://www.hiv.lanl.gov/components/sequence/HIV/asearch/query_one.comp?se_id=149247) Bottom of Form | Top of Form  [AJ389764](http://www.hiv.lanl.gov/components/sequence/HIV/asearch/query_one.comp?se_id=226286)Bottom of Form | AJ270000 | Top of Form  [AJ389765](http://www.hiv.lanl.gov/components/sequence/HIV/asearch/query_one.comp?se_id=226286) Bottom of Form | CM.AY371138 | CM.AY371138 |
|  | Top of Form  [AJ270002](http://www.hiv.lanl.gov/components/sequence/HIV/asearch/query_one.comp?se_id=149246) Bottom of Form | Top of Form  [AJ389765](http://www.hiv.lanl.gov/components/sequence/HIV/asearch/query_one.comp?se_id=226286) Bottom of Form | AJ270002 | Top of Form  [AJ389766](http://www.hiv.lanl.gov/components/sequence/HIV/asearch/query_one.comp?se_id=226286)Bottom of Form | CM.AY371142 | CM.AY371142 |
|  | Top of Form  [AJ270010](http://www.hiv.lanl.gov/components/sequence/HIV/asearch/query_one.comp?se_id=149241) Bottom of Form | Top of Form  [AJ389766](http://www.hiv.lanl.gov/components/sequence/HIV/asearch/query_one.comp?se_id=226286)Bottom of Form | AJ270010 | Top of Form  [AJ389767](http://www.hiv.lanl.gov/components/sequence/HIV/asearch/query_one.comp?se_id=226286)Bottom of Form | CM.AY371125 | CM.AY371125 |
|  | Top of Form  [AJ270014](http://www.hiv.lanl.gov/components/sequence/HIV/asearch/query_one.comp?se_id=149238) Bottom of Form | Top of Form  [AJ389767](http://www.hiv.lanl.gov/components/sequence/HIV/asearch/query_one.comp?se_id=226286)Bottom of Form | AJ270014 | Top of Form  [AJ389768](http://www.hiv.lanl.gov/components/sequence/HIV/asearch/query_one.comp?se_id=226286) Bottom of Form | CM.AY371126 | CM.AY371126 |
|  | Top of Form  [DD409979](http://www.hiv.lanl.gov/components/sequence/HIV/asearch/query_one.comp?se_id=81918) Bottom of Form | Top of Form  [AJ389768](http://www.hiv.lanl.gov/components/sequence/HIV/asearch/query_one.comp?se_id=226286)Bottom of Form | DQ168577 | Top of Form  [AJ389770](http://www.hiv.lanl.gov/components/sequence/HIV/asearch/query_one.comp?se_id=226286) Bottom of Form | CM.AY371127 | CM.AY371127 |
|  | Top of Form  [DQ168577](http://www.hiv.lanl.gov/components/sequence/HIV/asearch/query_one.comp?se_id=114230)Bottom of Form | Top of Form  [AJ389770](http://www.hiv.lanl.gov/components/sequence/HIV/asearch/query_one.comp?se_id=226286) Bottom of Form | DQ168578 | Top of Form  [AJ389771](http://www.hiv.lanl.gov/components/sequence/HIV/asearch/query_one.comp?se_id=226286)Bottom of Form | CM.AY371139 | CM.AY371139 |
|  | Top of Form  [L39106](http://www.hiv.lanl.gov/components/sequence/HIV/asearch/query_one.comp?se_id=183900) Bottom of Form | Top of Form  [AJ389771](http://www.hiv.lanl.gov/components/sequence/HIV/asearch/query_one.comp?se_id=226286) Bottom of Form | Top of Form  [L39106](http://www.hiv.lanl.gov/components/sequence/HIV/asearch/query_one.comp?se_id=183900) Bottom of Form | Top of Form  [AJ389777](http://www.hiv.lanl.gov/components/sequence/HIV/asearch/query_one.comp?se_id=226286)Bottom of Form | CM.AY371140 | CM.AY371140 |
|  |  | Top of Form  [AJ389777](http://www.hiv.lanl.gov/components/sequence/HIV/asearch/query_one.comp?se_id=226286)Bottom of Form |  | Top of Form  [DQ168577](http://www.hiv.lanl.gov/components/sequence/HIV/asearch/query_one.comp?se_id=114230)Bottom of Form | CM.AY371146 | CM.AY371146 |
|  |  | Top of Form  [DQ168577](http://www.hiv.lanl.gov/components/sequence/HIV/asearch/query_one.comp?se_id=114230)Bottom of Form |  | DQ168578 | CM.AY371128 | CM.AY371128 |
|  |  | DQ168578 |  | Top of Form  [L39106](http://www.hiv.lanl.gov/components/sequence/HIV/asearch/query_one.comp?se_id=183900)Bottom of Form | CM.AY371129 | CM.AY371129 |
|  |  | Top of Form  [L39106](http://www.hiv.lanl.gov/components/sequence/HIV/asearch/query_one.comp?se_id=183900) Bottom of Form |  | Top of Form  [U48628](http://www.hiv.lanl.gov/components/sequence/HIV/asearch/query_one.comp?se_id=250871) Bottom of Form | CM.AY371130 | CM.AY371130 |
|  |  | Top of Form  [U48628](http://www.hiv.lanl.gov/components/sequence/HIV/asearch/query_one.comp?se_id=250871) Bottom of Form |  |  | CM.AY371141 | CM.AY371141 |
|  |  |  |  |  | CM.AJ286133 | CM.AJ286133 |
|  |  |  |  |  | CM.AF377954 | CM.AF377954 |
|  |  |  |  |  | CM.AF377955 | CM.AF377955 |
|  |  |  |  |  | CM.AY271690 | CM.AY271690 |
|  |  |  |  |  | EC.AY151001 | EC.AY151001 |
|  |  |  |  |  | ES.EU786671 | ES.EU786671 |
|  |  |  |  |  | ES.EU884501 | ES.EU884501 |
|  |  |  |  |  | FR.AF063223 | FR.AF063223 |
|  |  |  |  |  | GH.AB286855 | GH.AB286855 |
|  |  |  |  |  | GH.AB286857 | GH.AB286857 |
|  |  |  |  |  | GH.AB286862 | GH.AB286862 |
|  |  |  |  |  | GH.AB286863 | GH.AB286863 |
|  |  |  |  |  | GH.AB231895 | GH.AB231895 |
|  |  |  |  |  | GH.AB231896 | GH.AB231896 |
|  |  |  |  |  | GH.AB231898 | GH.AB231898 |
|  |  |  |  |  | GH.AB049811 | GH.AB049811 |
|  |  |  |  |  | SE.AF107770 | SE.AF107770 |
|  |  |  |  |  | SN.AJ251056 | SN.AJ251056 |
|  |  |  |  |  | SN.AJ251057 | SN.AJ251057 |
|  |  |  |  |  | US.AY444811 | US.AY444811 |
|  |  |  |  |  | US.AY444809 | US.AY444809 |
|  |  |  |  |  | UZ.AY829214 | UZ.AY829214 |
| Subtype G | Top of Form  [AJ269980](http://www.hiv.lanl.gov/components/sequence/HIV/asearch/query_one.comp?se_id=149259)Bottom of Form | Top of Form  [AF069935](http://www.hiv.lanl.gov/components/sequence/HIV/asearch/query_one.comp?se_id=233535)Bottom of Form | Top of Form  [AJ269980](http://www.hiv.lanl.gov/components/sequence/HIV/asearch/query_one.comp?se_id=149259) Bottom of Form | Top of Form  [AF069935](http://www.hiv.lanl.gov/components/sequence/HIV/asearch/query_one.comp?se_id=233535)Bottom of Form | BE.AF084936 | BE.AF084936 |
|  | Top of Form  [AJ269995](http://www.hiv.lanl.gov/components/sequence/HIV/asearch/query_one.comp?se_id=149251)Bottom of Form | Top of Form  [AF069937](http://www.hiv.lanl.gov/components/sequence/HIV/asearch/query_one.comp?se_id=233535) Bottom of Form | Top of Form  [AJ269995](http://www.hiv.lanl.gov/components/sequence/HIV/asearch/query_one.comp?se_id=149251) Bottom of Form | Top of Form  [AF069937](http://www.hiv.lanl.gov/components/sequence/HIV/asearch/query_one.comp?se_id=233535) Bottom of Form | CM.AY371121 | CM.AY371121 |
|  | Top of Form  [AJ269996](http://www.hiv.lanl.gov/components/sequence/HIV/asearch/query_one.comp?se_id=149250)Bottom of Form | Top of Form  [AF069943](http://www.hiv.lanl.gov/components/sequence/HIV/asearch/query_one.comp?se_id=233527)Bottom of Form | Top of Form  [AJ269996](http://www.hiv.lanl.gov/components/sequence/HIV/asearch/query_one.comp?se_id=149250)Bottom of Form | Top of Form  [AF069943](http://www.hiv.lanl.gov/components/sequence/HIV/asearch/query_one.comp?se_id=233527)Bottom of Form | CM.AY772535 | CM.AY772535 |
|  | Top of Form  [AJ270003](http://www.hiv.lanl.gov/components/sequence/HIV/asearch/query_one.comp?se_id=149245) Bottom of Form | Top of Form  [AF069947](http://www.hiv.lanl.gov/components/sequence/HIV/asearch/query_one.comp?se_id=233527)Bottom of Form | Top of Form  [AJ270003](http://www.hiv.lanl.gov/components/sequence/HIV/asearch/query_one.comp?se_id=149245)Bottom of Form | Top of Form  [AF069947](http://www.hiv.lanl.gov/components/sequence/HIV/asearch/query_one.comp?se_id=233527)Bottom of Form | CU.AY586547 | CU.AY586547 |
|  | Top of Form  [AJ270011](http://www.hiv.lanl.gov/components/sequence/HIV/asearch/query_one.comp?se_id=226323) Bottom of Form | Top of Form  [AJ389723](http://www.hiv.lanl.gov/components/sequence/HIV/asearch/query_one.comp?se_id=226321)Bottom of Form | Top of Form  [AJ270011](http://www.hiv.lanl.gov/components/sequence/HIV/asearch/query_one.comp?se_id=226323) Bottom of Form | Top of Form  [AJ389723](http://www.hiv.lanl.gov/components/sequence/HIV/asearch/query_one.comp?se_id=226321)Bottom of Form | CU.AY586548 | CU.AY586548 |
|  | Top of Form  [AJ270012](http://www.hiv.lanl.gov/components/sequence/HIV/asearch/query_one.comp?se_id=149240)Bottom of Form | Top of Form  [AJ389724](http://www.hiv.lanl.gov/components/sequence/HIV/asearch/query_one.comp?se_id=226321)Bottom of Form | Top of Form  [AJ270012](http://www.hiv.lanl.gov/components/sequence/HIV/asearch/query_one.comp?se_id=149240) Bottom of Form | Top of Form  [AJ389724](http://www.hiv.lanl.gov/components/sequence/HIV/asearch/query_one.comp?se_id=226321) Bottom of Form | CU.AY586549 | CU.AY586549 |
|  | Top of Form  [DQ168573](http://www.hiv.lanl.gov/components/sequence/HIV/asearch/query_one.comp?se_id=114234) Bottom of Form | Top of Form  [AJ389725](http://www.hiv.lanl.gov/components/sequence/HIV/asearch/query_one.comp?se_id=226321)Bottom of Form | Top of Form  [DQ168573](http://www.hiv.lanl.gov/components/sequence/HIV/asearch/query_one.comp?se_id=114234)Bottom of Form | Top of Form  [AJ389725](http://www.hiv.lanl.gov/components/sequence/HIV/asearch/query_one.comp?se_id=226321) Bottom of Form | ES.EU786670 | ES.EU786670 |
|  | Top of Form  [DQ168575](http://www.hiv.lanl.gov/components/sequence/HIV/asearch/query_one.comp?se_id=114232)Bottom of Form | Top of Form  [AJ389726](http://www.hiv.lanl.gov/components/sequence/HIV/asearch/query_one.comp?se_id=226321)Bottom of Form | Top of Form  [DQ168575](http://www.hiv.lanl.gov/components/sequence/HIV/asearch/query_one.comp?se_id=114232) Bottom of Form | Top of Form  [AJ389726](http://www.hiv.lanl.gov/components/sequence/HIV/asearch/query_one.comp?se_id=226321) Bottom of Form | ES.AF450098 | ES.AF450098 |
|  | Top of Form  [DQ168576](http://www.hiv.lanl.gov/components/sequence/HIV/asearch/query_one.comp?se_id=114231)Bottom of Form | Top of Form  [AJ389727](http://www.hiv.lanl.gov/components/sequence/HIV/asearch/query_one.comp?se_id=226321) Bottom of Form | Top of Form  [DQ168576](http://www.hiv.lanl.gov/components/sequence/HIV/asearch/query_one.comp?se_id=114231) Bottom of Form | Top of Form  [AJ389727](http://www.hiv.lanl.gov/components/sequence/HIV/asearch/query_one.comp?se_id=226321) Bottom of Form | GH.AB287004 | GH.AB287004 |
|  | Top of Form  [DQ168579](http://www.hiv.lanl.gov/components/sequence/HIV/asearch/query_one.comp?se_id=114228) Bottom of Form | Top of Form  [AJ389728](http://www.hiv.lanl.gov/components/sequence/HIV/asearch/query_one.comp?se_id=226321) Bottom of Form | Top of Form  [DQ168579](http://www.hiv.lanl.gov/components/sequence/HIV/asearch/query_one.comp?se_id=114228)Bottom of Form | Top of Form  [AJ389728](http://www.hiv.lanl.gov/components/sequence/HIV/asearch/query_one.comp?se_id=226321)Bottom of Form | KE.AF061641 | KE.AF061641 |
|  | Top of Form  [U88826](http://www.hiv.lanl.gov/components/sequence/HIV/asearch/query_one.comp?se_id=240467) Bottom of Form | Top of Form  [AJ389729](http://www.hiv.lanl.gov/components/sequence/HIV/asearch/query_one.comp?se_id=226321)Bottom of Form | Top of Form  [U88826](http://www.hiv.lanl.gov/components/sequence/HIV/asearch/query_one.comp?se_id=240467) Bottom of Form | Top of Form  [AJ389729](http://www.hiv.lanl.gov/components/sequence/HIV/asearch/query_one.comp?se_id=226321) Bottom of Form | PT.AY612637 | PT.AY612637 |
|  |  | Top of Form  [AJ389730](http://www.hiv.lanl.gov/components/sequence/HIV/asearch/query_one.comp?se_id=226321)Bottom of Form |  | Top of Form  [AJ389730](http://www.hiv.lanl.gov/components/sequence/HIV/asearch/query_one.comp?se_id=226321) Bottom of Form | SE.AF061642 | SE.AF061642 |
|  |  | Top of Form  [AJ389731](http://www.hiv.lanl.gov/components/sequence/HIV/asearch/query_one.comp?se_id=226321)Bottom of Form |  | Top of Form  [AJ389731](http://www.hiv.lanl.gov/components/sequence/HIV/asearch/query_one.comp?se_id=226321) Bottom of Form |  |  |
|  |  | [AJ389732](http://www.hiv.lanl.gov/components/sequence/HIV/asearch/query_one.comp?se_id=226321) |  | [AJ389732](http://www.hiv.lanl.gov/components/sequence/HIV/asearch/query_one.comp?se_id=226321) |  |  |
|  |  | [AJ389733](http://www.hiv.lanl.gov/components/sequence/HIV/asearch/query_one.comp?se_id=226321) |  | [AJ389733](http://www.hiv.lanl.gov/components/sequence/HIV/asearch/query_one.comp?se_id=226321) |  |  |
|  |  | [AJ389734](http://www.hiv.lanl.gov/components/sequence/HIV/asearch/query_one.comp?se_id=226321) |  | [AJ389734](http://www.hiv.lanl.gov/components/sequence/HIV/asearch/query_one.comp?se_id=226321) |  |  |
|  |  | [AJ389735](http://www.hiv.lanl.gov/components/sequence/HIV/asearch/query_one.comp?se_id=226321) |  | [AJ389735](http://www.hiv.lanl.gov/components/sequence/HIV/asearch/query_one.comp?se_id=226321) |  |  |
|  |  | [AJ389737](http://www.hiv.lanl.gov/components/sequence/HIV/asearch/query_one.comp?se_id=226321) |  | [AJ389737](http://www.hiv.lanl.gov/components/sequence/HIV/asearch/query_one.comp?se_id=226321) |  |  |
|  |  | [AJ389738](http://www.hiv.lanl.gov/components/sequence/HIV/asearch/query_one.comp?se_id=226321) |  | [AJ389738](http://www.hiv.lanl.gov/components/sequence/HIV/asearch/query_one.comp?se_id=226321) |  |  |
|  |  | Top of Form  [AJ389740](http://www.hiv.lanl.gov/components/sequence/HIV/asearch/query_one.comp?se_id=226304)Bottom of Form |  | Top of Form  [AJ389740](http://www.hiv.lanl.gov/components/sequence/HIV/asearch/query_one.comp?se_id=226304) Bottom of Form |  |  |
|  |  | Top of Form  [AJ389742](http://www.hiv.lanl.gov/components/sequence/HIV/asearch/query_one.comp?se_id=226304) Bottom of Form |  | Top of Form  [AJ389742](http://www.hiv.lanl.gov/components/sequence/HIV/asearch/query_one.comp?se_id=226304) Bottom of Form |  |  |
|  |  | Top of Form  [AJ389743](http://www.hiv.lanl.gov/components/sequence/HIV/asearch/query_one.comp?se_id=226304) Bottom of Form |  | Top of Form  [AJ389743](http://www.hiv.lanl.gov/components/sequence/HIV/asearch/query_one.comp?se_id=226304) Bottom of Form |  |  |
|  |  | [AJ389744](http://www.hiv.lanl.gov/components/sequence/HIV/asearch/query_one.comp?se_id=226304) |  | [AJ389744](http://www.hiv.lanl.gov/components/sequence/HIV/asearch/query_one.comp?se_id=226304) |  |  |
|  |  | [AJ389745](http://www.hiv.lanl.gov/components/sequence/HIV/asearch/query_one.comp?se_id=226304) |  | [AJ389745](http://www.hiv.lanl.gov/components/sequence/HIV/asearch/query_one.comp?se_id=226304) |  |  |
|  |  | [AJ389746](http://www.hiv.lanl.gov/components/sequence/HIV/asearch/query_one.comp?se_id=226304) |  | [AJ389746](http://www.hiv.lanl.gov/components/sequence/HIV/asearch/query_one.comp?se_id=226304) |  |  |
|  |  | [AJ389747](http://www.hiv.lanl.gov/components/sequence/HIV/asearch/query_one.comp?se_id=226304) |  | [AJ389747](http://www.hiv.lanl.gov/components/sequence/HIV/asearch/query_one.comp?se_id=226304) |  |  |
|  |  | [AJ389748](http://www.hiv.lanl.gov/components/sequence/HIV/asearch/query_one.comp?se_id=226304) |  | [AJ389748](http://www.hiv.lanl.gov/components/sequence/HIV/asearch/query_one.comp?se_id=226304) |  |  |
|  |  | [AJ389749](http://www.hiv.lanl.gov/components/sequence/HIV/asearch/query_one.comp?se_id=226304) |  | [AJ389749](http://www.hiv.lanl.gov/components/sequence/HIV/asearch/query_one.comp?se_id=226304) |  |  |
|  |  | [AJ389750](http://www.hiv.lanl.gov/components/sequence/HIV/asearch/query_one.comp?se_id=226304) |  | [AJ389750](http://www.hiv.lanl.gov/components/sequence/HIV/asearch/query_one.comp?se_id=226304) |  |  |
|  |  | [AJ389751](http://www.hiv.lanl.gov/components/sequence/HIV/asearch/query_one.comp?se_id=226304) |  | [AJ389751](http://www.hiv.lanl.gov/components/sequence/HIV/asearch/query_one.comp?se_id=226304) |  |  |
|  |  | [AJ389752](http://www.hiv.lanl.gov/components/sequence/HIV/asearch/query_one.comp?se_id=226304) |  | [AJ389752](http://www.hiv.lanl.gov/components/sequence/HIV/asearch/query_one.comp?se_id=226304) |  |  |
|  |  | [AJ389753](http://www.hiv.lanl.gov/components/sequence/HIV/asearch/query_one.comp?se_id=226304) |  | [AJ389753](http://www.hiv.lanl.gov/components/sequence/HIV/asearch/query_one.comp?se_id=226304) |  |  |
|  |  | [AJ389754](http://www.hiv.lanl.gov/components/sequence/HIV/asearch/query_one.comp?se_id=226304) |  | [AJ389754](http://www.hiv.lanl.gov/components/sequence/HIV/asearch/query_one.comp?se_id=226304) |  |  |
|  |  | [AJ389755](http://www.hiv.lanl.gov/components/sequence/HIV/asearch/query_one.comp?se_id=226304) |  | [AJ389755](http://www.hiv.lanl.gov/components/sequence/HIV/asearch/query_one.comp?se_id=226304) |  |  |
|  |  | [AJ389756](http://www.hiv.lanl.gov/components/sequence/HIV/asearch/query_one.comp?se_id=226304) |  | [AJ389756](http://www.hiv.lanl.gov/components/sequence/HIV/asearch/query_one.comp?se_id=226304) |  |  |
|  |  | [AJ389757](http://www.hiv.lanl.gov/components/sequence/HIV/asearch/query_one.comp?se_id=226304) |  | [AJ389757](http://www.hiv.lanl.gov/components/sequence/HIV/asearch/query_one.comp?se_id=226304) |  |  |
|  |  | Top of Form  [DQ168573](http://www.hiv.lanl.gov/components/sequence/HIV/asearch/query_one.comp?se_id=114234) Bottom of Form |  | Top of Form  [DQ168573](http://www.hiv.lanl.gov/components/sequence/HIV/asearch/query_one.comp?se_id=114234)Bottom of Form |  |  |
|  |  | Top of Form  [DQ168575](http://www.hiv.lanl.gov/components/sequence/HIV/asearch/query_one.comp?se_id=114234)Bottom of Form |  | Top of Form  [DQ168575](http://www.hiv.lanl.gov/components/sequence/HIV/asearch/query_one.comp?se_id=114234)Bottom of Form |  |  |
|  |  | Top of Form  [DQ168576](http://www.hiv.lanl.gov/components/sequence/HIV/asearch/query_one.comp?se_id=114234)Bottom of Form |  | Top of Form  [DQ168576](http://www.hiv.lanl.gov/components/sequence/HIV/asearch/query_one.comp?se_id=114234)Bottom of Form |  |  |
|  |  | Top of Form  [DQ168579](http://www.hiv.lanl.gov/components/sequence/HIV/asearch/query_one.comp?se_id=114234)Bottom of Form |  | Top of Form  [DQ168579](http://www.hiv.lanl.gov/components/sequence/HIV/asearch/query_one.comp?se_id=114234)Bottom of Form |  |  |
|  |  | Top of Form  [U88826](http://www.hiv.lanl.gov/components/sequence/HIV/asearch/query_one.comp?se_id=240467) Bottom of Form |  | Top of Form  [U88826](http://www.hiv.lanl.gov/components/sequence/HIV/asearch/query_one.comp?se_id=240467) Bottom of Form |  |  |
| Others | Top of Form  [AF212292](http://www.hiv.lanl.gov/components/sequence/HIV/asearch/query_one.comp?se_id=68688)Bottom of Form | Top of Form  [AF069934](http://www.hiv.lanl.gov/components/sequence/HIV/asearch/query_one.comp?se_id=233536) Bottom of Form |  |  |  |  |
|  | Top of Form  [AJ245483](http://www.hiv.lanl.gov/components/sequence/HIV/asearch/query_one.comp?se_id=149269) Bottom of Form | Top of Form  [AJ245484](http://www.hiv.lanl.gov/components/sequence/HIV/asearch/query_one.comp?se_id=217298)Bottom of Form |  |  |  |  |
|  | Top of Form  [AJ270004](http://www.hiv.lanl.gov/components/sequence/HIV/asearch/query_one.comp?se_id=149244)Bottom of Form | Top of Form  [AJ389741](http://www.hiv.lanl.gov/components/sequence/HIV/asearch/query_one.comp?se_id=226303)Bottom of Form |  |  |  |  |
|  | Top of Form  [AJ270013](http://www.hiv.lanl.gov/components/sequence/HIV/asearch/query_one.comp?se_id=149239) Bottom of Form | Top of Form  [AJ389755](http://www.hiv.lanl.gov/components/sequence/HIV/asearch/query_one.comp?se_id=226303)Bottom of Form |  |  |  |  |
|  | Top of Form  [AJ269979](http://www.hiv.lanl.gov/components/sequence/HIV/asearch/query_one.comp?se_id=226336)Bottom of Form | Top of Form  [AJ389762](http://www.hiv.lanl.gov/components/sequence/HIV/asearch/query_one.comp?se_id=226282)Bottom of Form |  |  |  |  |
|  | Top of Form  [AJ269981](http://www.hiv.lanl.gov/components/sequence/HIV/asearch/query_one.comp?se_id=226335) Bottom of Form | Top of Form  [AJ389773](http://www.hiv.lanl.gov/components/sequence/HIV/asearch/query_one.comp?se_id=226271) Bottom of Form |  |  |  |  |
|  | Top of Form  [AJ269983](http://www.hiv.lanl.gov/components/sequence/HIV/asearch/query_one.comp?se_id=226335) Bottom of Form | Top of Form  [AJ389774](http://www.hiv.lanl.gov/components/sequence/HIV/asearch/query_one.comp?se_id=226271) Bottom of Form |  |  |  |  |
|  | Top of Form  [AJ269984](http://www.hiv.lanl.gov/components/sequence/HIV/asearch/query_one.comp?se_id=226333) Bottom of Form | Top of Form  [AJ389776](http://www.hiv.lanl.gov/components/sequence/HIV/asearch/query_one.comp?se_id=226271) Bottom of Form |  |  |  |  |
|  | Top of Form  [AJ269986](http://www.hiv.lanl.gov/components/sequence/HIV/asearch/query_one.comp?se_id=226332)Bottom of Form | Top of Form  [AJ389778](http://www.hiv.lanl.gov/components/sequence/HIV/asearch/query_one.comp?se_id=226271) Bottom of Form |  |  |  |  |
|  | Top of Form  [AJ269989](http://www.hiv.lanl.gov/components/sequence/HIV/asearch/query_one.comp?se_id=226331) Bottom of Form | Top of Form  [AJ389779](http://www.hiv.lanl.gov/components/sequence/HIV/asearch/query_one.comp?se_id=226271)Bottom of Form |  |  |  |  |
|  | Top of Form  [AJ269990](http://www.hiv.lanl.gov/components/sequence/HIV/asearch/query_one.comp?se_id=226330) Bottom of Form | Top of Form  [AF069932](http://www.hiv.lanl.gov/components/sequence/HIV/asearch/query_one.comp?se_id=233538)Bottom of Form |  |  |  |  |
|  | Top of Form  [AJ270005](http://www.hiv.lanl.gov/components/sequence/HIV/asearch/query_one.comp?se_id=226326) Bottom of Form | Top of Form  [AF069939](http://www.hiv.lanl.gov/components/sequence/HIV/asearch/query_one.comp?se_id=233538)Bottom of Form |  |  |  |  |
|  | Top of Form  [AJ270006](http://www.hiv.lanl.gov/components/sequence/HIV/asearch/query_one.comp?se_id=149243) Bottom of Form | Top of Form  [FJ977087](http://www.hiv.lanl.gov/components/sequence/HIV/asearch/query_one.comp?se_id=283210)Bottom of Form |  |  |  |  |
|  | Top of Form  [AJ270007](http://www.hiv.lanl.gov/components/sequence/HIV/asearch/query_one.comp?se_id=226325) Bottom of Form | Top of Form  [FJ977092](http://www.hiv.lanl.gov/components/sequence/HIV/asearch/query_one.comp?se_id=283210) Bottom of Form |  |  |  |  |
|  | Top of Form  [AJ270008](http://www.hiv.lanl.gov/components/sequence/HIV/asearch/query_one.comp?se_id=226325)Bottom of Form | Top of Form  [FJ977093](http://www.hiv.lanl.gov/components/sequence/HIV/asearch/query_one.comp?se_id=283210)Bottom of Form |  |  |  |  |
|  | Top of Form  [AJ270009](http://www.hiv.lanl.gov/components/sequence/HIV/asearch/query_one.comp?se_id=226325) Bottom of Form | Top of Form  [FJ977095](http://www.hiv.lanl.gov/components/sequence/HIV/asearch/query_one.comp?se_id=283210)Bottom of Form |  |  |  |  |
|  | Top of Form  [AJ270015](http://www.hiv.lanl.gov/components/sequence/HIV/asearch/query_one.comp?se_id=226325) Bottom of Form | Top of Form  [AF069945](http://www.hiv.lanl.gov/components/sequence/HIV/asearch/query_one.comp?se_id=233525)Bottom of Form |  |  |  |  |
|  | Top of Form  [DQ168574](http://www.hiv.lanl.gov/components/sequence/HIV/asearch/query_one.comp?se_id=114233)Bottom of Form | Top of Form  [AJ389780](http://www.hiv.lanl.gov/components/sequence/HIV/asearch/query_one.comp?se_id=226264) Bottom of Form |  |  |  |  |
|  | Top of Form  [AJ269985](http://www.hiv.lanl.gov/components/sequence/HIV/asearch/query_one.comp?se_id=149257)Bottom of Form | Top of Form  [AJ389781](http://www.hiv.lanl.gov/components/sequence/HIV/asearch/query_one.comp?se_id=226263) Bottom of Form |  |  |  |  |
|  | Top of Form  [AJ269991](http://www.hiv.lanl.gov/components/sequence/HIV/asearch/query_one.comp?se_id=149254) Bottom of Form | Top of Form  [AJ389775](http://www.hiv.lanl.gov/components/sequence/HIV/asearch/query_one.comp?se_id=226269)Bottom of Form |  |  |  |  |
|  | Top of Form  [AJ269994](http://www.hiv.lanl.gov/components/sequence/HIV/asearch/query_one.comp?se_id=226329) Bottom of Form | Top of Form  [U88825](http://www.hiv.lanl.gov/components/sequence/HIV/asearch/query_one.comp?se_id=233870) Bottom of Form |  |  |  |  |
|  | Top of Form  [U88825](http://www.hiv.lanl.gov/components/sequence/HIV/asearch/query_one.comp?se_id=233870) Bottom of Form |  |  |  |  |  |
|  | Top of Form  [AJ270001](http://www.hiv.lanl.gov/components/sequence/HIV/asearch/query_one.comp?se_id=226327) Bottom of Form |  |  |  |  |  |

* All the available Nigerian sequences (corresponding to the coordinates of our sequences) downloaded from the HIV Los Alamos data base to determine the relationship (figure 2) of study isolates to previous Nigerian HIV-1 isolates.

** All the available dated Nigerian sequences (corresponding to the coordinates of our sequences) downloaded from the HIV Los Alamos data base to determine the relationship (figure 4) between Nigerian isolates and reference sequences from other parts of the World.

*** The reference sequences (figure 4) from other countries downloaded from the HIV Los Alamos data base. The accession numbers are preceded by two letter code of the sampling country - BE: Belgium; CM: Cameroon; EC: Ecuador; ES: Spain; FR: France; GH: Ghana; KE: Kenya; PT: Portugal; SE: Sweden; SN: Senegal; US: United States; UZ: Uzbekistan.
