# Supplementary material for: Genetic Characteristics, Coreceptor Usage Potential and Evolution of Nigerian HIV-1 Subtype G and CRF02_AG Isolates
Source: PLoS One. 2011 Mar 14;6(3):e17865. doi: 10.1371/journal.pone.0017865 (PMC3056731; doi:10.1371/journal.pone.0017865)
Supplement: Table S2 — Date of Most Common Recent Ancestor (MRCA) and parameters estimated from BEAST. (DOC) [file pone.0017865.s002.doc]

**Table S2. Date of Most Common Recent Ancestor (MRCA) and parameters estimated from BEAST.**

| **Dataset** | **Models** | **Mean** | **STDEV of Mean** | **Median** | **95% HPD lower** | **95% HPD upper** | **ACT** | **ESS** |
| --- | --- | --- | --- | --- | --- | --- | --- | --- |
| HIV-1G GAG | Constant Strict | 1955.62 | 1.78 | 1963.68 | 1981.97 | 1920.8 | 12325.16 | 2921.67 |
|  | Constant Relax | 1941.01 | 2.01 | 1957.26 | 1982.87 | 1867.4 | 18231.31 | 1975.17 |
|  | Exponential Strict | 1971.94 | 0.34 | 1973.87 | 1984.11 | 1956.26 | 50385.94 | 714.68 |
|  | Exponential Relax | 1974.78 | 0.8 | 1977.34 | 1988.06 | 1959.43 | 136710 | 263.4 |
|  | BSP Strict | 1971.38 | 0.21 | 1972.86 | 1982.29 | 1957.47 | 22245.17 | 1504.6 |
|  | BSP Relax | 1970.67 | 0.32 | 1973.22 | 1984.93 | 1953.41 | 20037 | 1797.17 |
| HIV1-G ENV | Constant Strict | 1968.7 | 0.09 | 1969.25 | 1976.12 | 1960.3 | 12820.61 | 4212.75 |
|  | Constant Relax | 1968.61 | 0.14 | 1969.54 | 1979.28 | 1955.59 | 24022.38 | 2248.32 |
|  | Exponential Strict | 1967.9 | 4.92 | 1973.16 | 1979.04 | 1965.6 | 573240 | 94.22 |
|  | Exponential Relax | 1098.2 | 710.96 | 1970.96 | 1986.05 | -1483.81 | 8555700 | 3.16 |
|  | BSP Strict | 1969.29 | 0.07 | 1969.79 | 1976.29 | 1961.21 | 13580.3 | 3977.08 |
|  | BSP Relax | 1970.86 | 0.11 | 1971.6 | 1980.28 | 1960.11 | 18812.02 | 2871.04 |

STDEV: standard deviation; ACT: auto-correlation time; ESS: effective sample size.
