# Supplementary material for: Genetic Characteristics, Coreceptor Usage Potential and Evolution of Nigerian HIV-1 Subtype G and CRF02_AG Isolates
Source: PLoS One. 2011 Mar 14;6(3):e17865. doi: 10.1371/journal.pone.0017865 (PMC3056731; doi:10.1371/journal.pone.0017865)
Supplement: Table S3 — Evolutionary rate and parameters estimated from BEAST. (DOC) [file pone.0017865.s003.doc]

**Table S**3. Evolutionary rate and parameters estimated from BEAST.

| **Dataset** | **Models** | **Mean** | **STDEV of Mean** | **Median** | **95% HPD lower** | **95% HPD upper** | **ACT** | **ESS** |
| --- | --- | --- | --- | --- | --- | --- | --- | --- |
| HIV-1G GAG | Constant Strict | 1.74E-03 | 1.26E-05 | 1.74E-03 | 6.56E-04 | 2.76E-03 | 19145.8 | 1880.83 |
|  | Constant Relax | 2.19E-03 | 3.10E-05 | 2.10E-03 | 4.83E-04 | 3.99E-03 | 35981.05 | 1000.8 |
|  | Exponential Strict | 1.96E-03 | 1.41E-05 | 1.96E-03 | 1.12E-03 | 2.84E-03 | 37040.28 | 972.18 |
|  | Exponential Relax | 2.35E-03 | 3.85E-05 | 2.30E-03 | 1.05E-03 | 3.88E-03 | 102000 | 353.6 |
|  | BSP Strict | 2.12E-03 | 9.92E-06 | 2.11E-03 | 1.29E-03 | 2.94E-03 | 18371.02 | 1821.89 |
|  | BSP Relax | 2.29E-03 | 1.37E-05 | 2.29E-03 | 1.15E-03 | 3.44E-03 | 19622.22 | 1835.16 |
| HIV-1G ENV | Constant Strict | 9.45E-03 | 2.79E-05 | 9.37E-03 | 6.37E-03 | 1.24E-02 | 16973.22 | 3182.07 |
|  | Constant Relax | 1.02E-02 | 5.95E-05 | 1.01E-02 | 6.13E-03 | 1.49E-02 | 36675.1 | 1472.66 |
|  | Exponential Strict | 8.50E-03 | 1.04E-04 | 8.46E-03 | 5.95E-03 | 1.17E-02 | 207000 | 260.74 |
|  | Exponential Relax | 5.59E-03 | 2.57E-03 | 7.14E-03 | 3.21E-05 | 1.22E-02 | 8320000 | 3.25 |
|  | BSP Strict | 8.52E-03 | 2.57E-05 | 8.42E-03 | 6.02E-03 | 1.14E-02 | 17963.36 | 3006.68 |
|  | BSP Relax | 9.13E-03 | 5.03E-05 | 9.01E-03 | 5.50E-03 | 1.30E-02 | 35962.43 | 1501.85 |

STDEV: standard deviation; ACT: auto-correlation time; ESS: effective sample size.
